# Optical coherence tomography angiography: microvascular alterations in diabetic eyes without diabetic retinopathy

**DOI:** 10.5935/0004-2749.20210023

**Published:** 2025-02-02

**Authors:** Cristiana Lumack do Monte Agra, Rodrigo Pessoa Cavalcanti Lira, Fernanda Galvão Pinheiro, Larissa Halley Soares e Sá, Vasco Torres Fernandes Bravo Filho

**Affiliations:** 1 Department of Retina and Vitreous, Fundação Altino Ventura, Recife, PE, Brazil; 2 Universidade Federal de Pernambuco, Recife, PE, Brazil; 3 Department of Ophthalmology, Fundação Altino Ventura, Recife, PE, Brazil

**Keywords:** Angiography, Diabetes mellitus, Diabetic retinopathy, Diagnostic imaging, Tomographpy, optical coherence, Angiografia, Diabetes mellitus, Retinopatia diabética, Diagnóatico por imagem, Tomografia de coerência óptica

## Abstract

**Purpose:**

To describe microvascular changes in the maculas of individuals with type 2 diabetes observed on optical coherence tomography angiography (OCTA) images. We compared the maculas of diabetic subjects without diabetic retinopathy with those of healthy subjects and correlated the findings with the clinical profiles of diabetic subjects.

**Methods:**

One eye each of 30 patients with diabetes and 30 healthy individuals were examined. The patients with diabetes underwent funduscopy, retinography, and fluorescein angiography to rule out retinopathy. All subjects underwent optical coherence tomography angiography of a macular area (6×6 mm^2^), and the foveal and parafoveal vascular densities were analyzed in the superficial and deep retinal vascular plexus. The foveal and parafoveal thicknesses, foveal avascular zone of the superficial plexus, and choriocapillaris flow area were also examined. The optical coherence tomography angiography results were compared between the two study groups and correlated with the following parameters: visual acuity, time since diabetes diagnosis, glycemic control, lipid profile, and renal function of patients with diabetes.

**Results:**

A minimal increase in the choriocapillaris flow area was observed in the patients with diabetes (mean area, 22.3 ± 4.6 mm^2^ in controls; 22.6 ± 3.9 mm^2^ in patients with diabetes) (p=0.017). No significant differences were observed between other optical coherence tomography angiography parameters analyzed in the two groups. Glycosylated hemoglobin and fasting blood glucose levels were significantly negatively correlated with the foveal vascular density of both plexuses; conversely, fasting blood glucose levels were positively correlated with the choriocapillaris flow area (p=0.034). The other clinical parameters were not correlated with the optical coherence tomography angiography findings.

**Conclusion:**

Optical coherence tomography angiography may not be the most appropriate tool for detecting preclinical changes in patients with diabetes, moreover, optical coherence tomography angiography; does not replace clinical examinations. Glycemic control should be the primary clinical parameter considered during retinopathy screening. Larger studies are necessary to confirm these findings.

## Abstract (pt)

**Objetivo:**

Descrever alterações microvasculares na mácula em diabéticos do tipo 2 sem retinopatia diabética e pacientes saudáveis, e correlacionar achados com perfil clínico nos diabéticos.

**Métodos:**

Foram incluídos 60 olhos de 30 diabéticos e 30 pacientes saudáveis. Diabéticos realizaram fundoscopia, retinografia^®^ (CR2; Canon Inc., New York, New York, USA) e an giografia fluoresceínica^®^ (TRC-50DXC; Topcon Inc., Tokyo, Japan) para descartar a presença de retinopatia. Os 60 pacientes realizaram a angiografia por tomografia de coerência óptica^®^ (RTVue XR, Avanti, Optovue, Fremont, CA, USA) (área macular: 6 x 6 mm^2^) e foram analisados densidade vascular total, foveal e parafoveal no plexo capilar superficial e plexo capilar profundo, espessura foveal, espessura parafoveal, área da zona avascular da fóvea no plexo capilar superficial e área de fluxo da coriocapilar. Resultados da angiografia por tomografia de coerência óptica foram comparados entre os 2 grupos e correlacionados com acuidade visual, tempo de diabetes, controle glicêmico, perfil lipídico e função renal nos diabéticos.

**Resultados:**

Observou-se aumento mínimo da área de fluxo da coriocapilar nos diabéticos, média das áreas foi de 22,3 ± 4,6 mm^2^ no grupo controle e 22,6 ± 3,9 mm^2^ em diabéticos (p=0,017). Não foi observada diferença estatisticamente significante entre outras variáveis da angiografia por tomografia de coerência óptica analisadas nos dois grupos. Hemoglobina glicosilada e glicemia de jejum apresentaram correlação negativa estatisticamente significante com densidade vascular foveal de ambos os plexos e a glicemia de jejum se correlacionou positivamente com área de fluxo da coriocapilar (p=0,034). Outros dados clínicos avaliados não apresentaram correlação com achados da angiografia por tomografia de coerência óptica.

**Conclusão:**

Resultados sugerem que a angiografia por tomografia de coerência óptica pode não ser a melhor ferramenta na detecção de alterações pré-clínicas em diabéticos, não substituindo o exame clínico, e corroboram a ideia de que o controle glicêmico deve ser o principal parâmetro clínico a ser considerado na triagem da retinopatia. Estudos com amostras maiores são necessários para confirmar os achados.

## INTRODUCTION

Diabetes mellitus (DM) has emerged as a serious glo bal public health issue. In the United States alone, 8 million individuals have been diagnosed with diabetes, primarily with type 2 diabetes, and the prevalence of DM in Brazil is comparable to that of the US^(1,2)^. Diabetic retinopathy (DR) is the leading cause of blindness in economically developed populations. DR affects approximately 75% of patients with DM 15 years after disease onset^(3)^.

Despite its sensitivity in detecting the early changes associated with DR, including microaneurysms and increased retinal capillary permeability, fluorescein angiography (FA) is not clinically indicated to screen for early nonproliferative DR given its possible associated side effects^(4)^. Optical coherence tomography angiography (OCTA), a more recent technology, can rapidly and noninvasively map the retinal and choroidal microvasculature. This technique enables the separation of the retinal capillary plexuses, provides reliable quantitative information, and has shown promise for detecting vascular changes in DR^(5,6)^. Researchers have investigated the use of OCTA in DR by quantifying areas of nonperfusion and vascular density (VD) and by evaluating the foveal avascular zone (FAZ), changes in the choriocapillaris (CC), and other parameters^(7-10)^.

The current study aimed to analyze and quantify the vascular changes in the macula in patients with type 2 DM without signs of DR by using OCTA. This study also evaluated the ability OCTA to detect preclinical changes, such as retinopathy onset, and to correlate the findings with clinical data, such as glucose levels, lipid profiles, and renal function.

## METHODS

This cross-sectional study was conducted from March 2017 to December 2018 and included 30 subjects (30 eyes) with type 2 DM without DR; the absence of DR was ruled out via a clinical examination, retinography, and FA. A control group of 30 individuals (30 eyes) without diabetes was also included. The study subjects were matched for age and the presence of systemic arterial hypertension. The participants were recruited from the Hospital das Clínicas, the Hospital Agamenom, and the Maternal Infant Institute from among individuals with a previous diagnosis of diabetes that had been determined at the respective endocrinology reference services and who were referred for evaluation at the Fundação Altino Ventura in Recife, Brazil. The exclusion criteria for both groups included a history of vitreoretinal surgery, photocoagulation, glaucoma, a refractive error exceeding -6.00 or +6.00 diopters, optical media opacities, and other chorioretinal disorders, such as vascular diseases, epiretinal membranes, or vitreomacular traction.

The following data were collected from the subjects with diabetes: gender, age, time since DM diagnosis, presence of comorbidities (e.g., systemic arterial hypertension and dyslipidemias), and the use of insulin. An ophthalmologic clinical examination included measurements of the participants’ best-corrected visual acuity (BCVA), presented as the logarithm of the minimum angle of resolution units (Early Treatment of Diabetic Retinopathy Study table), and a biomicroscopic evaluation under mydriasis of the anterior and posterior segments. In the absence of DR, funduscopy was performed using retinography (CR2, Canon, Inc., New York, NY, USA), followed by FA (TRC-50DXC, Topcon, Inc., Tokyo, Japan). The following laboratory measurements were obtained in diabetic subjects with normal FA findings: fasting blood glucose (FBG), glycosylated hemoglobin (HbA1c), total cholesterol, low-density lipoprotein, high-density lipoprotein, triglycerides, urea, creatinine levels, and the urinary albumin/creatinine ratio. The participants were considered to have dyslipidemia if they reported the use of medications for this condition and/or presented with laboratory findings consistent with the V Brazilian Guidelines on Dyslipidemias.

The control subjects and subjects with diabetes who did not have signs of DR during FA underwent OCTA (RTVue XR, Avanti, Optovue, Fremont, CA, USA) in the AngioRetina mode. The Angiovue system (version 2015.100.0.35) operates at 70,000 scans/second at a wavelength of 840 nm; it has tissue resolutions of 5 µm (axial) and 15 µm (transverse) and uses a split-spectrum amplitude decorrelation angiography algorithm to reduce noise and enhance image quality^(11)^. The assessed images were *en face* 6×6 mm area macular views centered on the fovea. The superficial capillary plexus (SCP) was delimited from 3 µm below the internal limiting membrane to approximately 15 µm below the inner margin of the internal plexiform. The deep capillary plexus (DCP) was comprised of the area 15 µm below the internal margin of the internal plexiform layer and 70 µm below the outer margin of the outer plexiform layer. The CC was comprised of an approximately 30-µm thick capillary layer posterior to the junction of the retinal pigment epithelium and Bruch’s membrane^(7,11)^. The software used provides a circular grid in which the center corresponds to the fovea on both the SCP and DCP maps and divides the foveal and parafoveal areas with inner and outer rings that are approximately 1.0 and 3.0 mm in diameter, respectively. The AngioAnalytics (Optovue) software program automatically measures the areas where blood flow is present (mm^2^) and calculates the VD (percentage)^(11)^. In the current study, we used the SCP and DCP values to determine the total macular VD (36 mm^2^) and both the foveal and parafoveal VDs. The FAZs in the SCP and CC flow area were calculated, and the foveal and parafoveal thicknesses (µm) were also measured.

The subjects with diabetes and the individuals in the control group each underwent OCTA three times; the means of the three values obtained for each variable were used for analysis. One eye from each participant was included in the study and was selected based on the availability of the highest quality images, as determined by two ophthalmologists. Cases with a signal strength index below 50, low-quality images secondary to poor fixation, and media opacities or artifacts, which were defined as the inability to delineate between the capillaries in at least one-third of the images, were excluded^(11,12)^.

The statistical analysis was performed using the Statistical Package for the Social Sciences (SPSS)^®^, version 25.0 (2017, IBM Corporation, Armonk, NY, USA). The quantitative variables were expressed as the means, standard deviation (SD), and maximal and minimal values. The qualitative variables were expressed in absolute and relative frequencies. Normality was assessed using the Shapiro-Wilk test, and the non-Gaussian distribution was determined. The nonparametric Mann-Whitney *U*-test was used to compare the numerical variables obtained between the study group and the control group using the AngioAnalytics software. Pearson’s correlation coefficient was used to evaluate the correlation between the AngioAnalytics parameters, the time since the DM diagnosis, and the laboratory results of those in the DM group. P<0.05 was considered significant.

The institutional research ethics committee (CEP/CONEP CAAE: 59457816.7.0000.5532) approved the study protocol, and all participants signed an informed consent form.

## RESULTS

Sixty eyes were evaluated in this study, which included 30 from patients with diabetes and 30 from subjects in the control group. Among the diabetic subjects, the mean time since the DM diagnosis was 73.4 ± 74.0 months; 4 (13.3%) subjects had renal failure, 21 (70.0%) had dyslipidemia, and 5 (16.7%) used insulin. The participants’ characteristics are shown in table 1.

**Table 1:** Participant characteristics

	Diabetics		Controls		*p* value
Age (years) (mean ± SD)	60.0 ± 8.0		60.0 ± 11.0		0.83^**^
Sex [n (%)]	Female: 20 (66.7%)		Female: 21 (70.7%)		0.781^*^
	Male: 10 (33.3%)		Male: 9 (30%)		
Evaluated eye [n (%)]	Right: 16 (53.3%)		Right: 15 (50%)		0.796^*^
	Left: 14 (46.7%)		Left: 15 (50%)		
Systemic arterial hypertension [n (%)]	Yes: 20 (66.7%)		Yes: 20 (66.7%)		1^*^
	No: 10 (33.3%)		No: 10 (33.3%)		
BCVA (logMAR) (mean ± SD)	0.1 ± 0.3		0.1 ± 0.2		0.812^**^
Pseudophakics [n (%)]	Yes: 2 (6.7%)		Yes: 5 (16.7%)		0.11^*^
	No: 28 (93.3%)		No: 25 (83.3%)		

SD= standard deviation; BCVA= best-corrected visual acuity.

* = Chi-square test;

** = Mann-Whitney *U*-test.

The laboratory test results reported that most (26 subjects, 86.7%) of the subjects with diabetes had HbA1c values exceeding 6%. Ten (33.3%) diabetic subjects had total cholesterol levels over 200 mg/dL and triglyceride levels over 150 mg/dL whereas only two (6.7%) had low-density lipoprotein values over 160 mg/dL and high-density lipoprotein levels below 40 mg/dL. Few (n=4; 13.3%) had a serum albumin/creatinine ratio greater than 30.0 mg/g. The laboratory test results are shown in table 2.

**Table 2:** Laboratory test results of diabetics patients: mean, SD, and maximum and minimum values

	n	Mean ± SD		Minimum value	Maximum value
FBG (mg/dL)	30	167.1 ± 65.0		91.0	302.5
HbA1C (%)	30	7.8 ± 2.5		0.6	12.6
Total Cholesterol (mg/dL)	30	193.5 ± 40.9		140.0	297.0
LDL (mg/dL)	30	110.4 ± 32.9		49.0	179.2
HDL (mg/dL)	30	57.1 ± 27.8		33.0	193.0
Triglycerides (mg/dL)	29	146.4 ± 74.7		54.0	339.0
Urea (mg/dL)	29	31.6 ± 8.6		16.7	55.0
Creatinine (mg/dL)	29	0.8 ± 0.2		0.4	1.3
Urine albumin/creatinine ratio (mg/g)	30	31.7 ± 91.6		0.0	446.0

SD= standard deviation; HbA1C= glycosylated hemoglobin.

**Figure 1:**
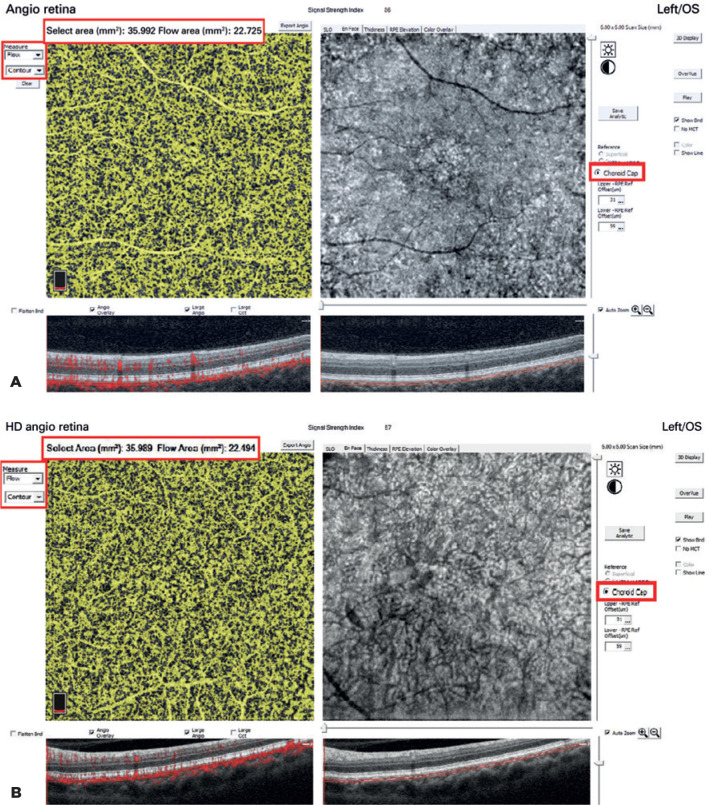
Examples of choriocapillaris maps (A, control subject; B, patient with diabetes). The flow area was calculated automatically (red box).

The mean CC flow area was 22.6 ± 3.9 mm^2^ in the diabetic group and 22.3 ± 4.6 mm^2^ in the control group (p=0.017) (Figure 1). No other significant differences were observed between the two groups. Table 3 shows the AngioAnalytics software findings obtained from both groups and a comparative evaluation.

**Table 3:** Angioanalytics results: comparison between diabetics and healthy subject

	Diabetics (n=30) mean ± SD	Controls (n=30) mean ± SD	*p* value
SSI	69.5 ± 6.5	71.1 ± 7.8	0.315^*^
Foveal thickness (µm)	242.9 ± 20.8	240.9 ± 21.4	0.756^*^
Parafoveal thickness (µm)	309.3 ± 17.0	341.6 ± 172.6	0.620^*^
CC flow area (mm^2^)	22.6 ± 3.9	22.3 ± 4.6	0.017^*^
FAZ area (mm^2^)	0.4 ± 0.1	0.4 ± 0.1	0.179^*^
Total SCP VD (%)	52.4 ± 3.2	51.2 ± 4.8	0.525^*^
Foveal SCP VD (%)	28.3 ± 5.9	27.7 ± 5.0	0.487^*^
Parafoveal SCP VD (%)	55.4 ± 3.8	55.4 ± 4.1	0.953^*^
Total DCP VD (%)	54.3 ± 3.2	53.1 ± 4.4	0.408^*^
Foveal DCP VD (%)	28.3 ± 6.4	28.2 ± 5.8	0.690^*^
Parafoveal SCP VD (%)	58.1 ± 3.6	57.5 ± 4.3	0.723^*^

SD= standard deviation; SSI= signal strength index; CC= choriocapillary; FAZ= foveal avascular zone; SCP= superficial capillary plexus; VD= vascular density; DCP= deep capillary plexus.

* = Mann-Whitney *U*-test.

No correlations were found between the AngioAnalytics results, the time since DM diagnosis, and the BCVA in the subjects with diabetes. An analysis of the laboratory test results showed correlations between the FBG and foveal thickness (p=0.006), CC flow area (p=0.034), superficial foveal VD (p=0.026), and deep foveal VD (p=0.017). Correlations were also observed between the HbA1c levels and the SCP foveal VD (p=0.035) and DCP foveal VD (p=0.016). No significant correlations were identified between the AngioAnalytics results and the participants’ specific lipid and renal profiles (Table 4).

**Table 4:** Analysis of pearson’s correlation between duration of diabetes, visual acuity, and laboratory test results versus findings of angioanalytics in diabetic subjects

		SSI	FT	PT	CC flow area		FAZ area		TSVD	FSVD	PSVD	TDVD	FDVD	PDVD
BCVA	**r**	-0.274	0.275	0.225	0.053		-0.087		-0.174	0.152	-0.208	-0.203	0.135	-0.250
(logMAR)	*p*	0.143	0.141	0.233	0.783		0.647		0.358	0.423	0.270	0.281	0.476	0.183
	**n**	30	30	30	30		30		30	30	30	30	30	30
Duration of DM (months)	**r** *p*	-0.144 0.458	-0.169 0.382	-0.006 0.975		-0.039 0.839		0.239 0.211	-0.092 0.636	-0.158 0.414	-0.024 0.903	-0.063 0.746	-0.142 0.461	0.003 0.986
	**n**	29	29	29		29		29	29	29	29	29	29	29
FBG	**r**	0.233	-0.490	-0.282		0.388		0.353	0.148	-0.405	0.196	0.181	-0.433	0.184
	*p*	0.214	0.006	0.132		0.034		0.056	0.436	0.026	0.299	0.338	0.017	0.331
	**n**	30	30	30		30		30	30	30	30	30	30	30
HbA1C	**r**	0.264	-0.304	-0.272		0.218		0.287	0.187	-0.387	0.228	0.237	-0.436	0.275
	*p*	0.159	0.103	0.145		0.247		0.124	0.323	0.035	0.226	0.207	0.016	0.142
	**n**	30	30	30		30		30	30	30	30	30	30	30
TC	**r**	0.188	0.224	0.188		0.088		-0.171	0.096	0.027	0.039	0.041	0.026	-0.018
	*p*	0.321	0.234	0.319		0.645		0.367	0.613	0.887	0.840	0.830	0.894	0.923
	**n**	30	30	30		30		30	30	30	30	30	30	30
LDL	**r**	0.298	0.234	0.216		0.230		-0.145	0.253	-0.001	0.223	0.202	-0.019	0.159
	*p*	0.110	0.214	0.252		0.222		0.445	0.177	0.997	0.236	0.285	0.922	0.402
	**n**	30	30	30		30		30	30	30	30	30	30	30
HDL	**r**	-0.220	0.056	-0.117		-0.031		-0.009	-0.327	0.085	-0.307	-0.357	0.084	-0.345
	*p*	0.243	0.770	0.537		0.869		0.962	0.078	0.654	0.099	0.053	0.660	0.062
	**n**	30	30	30		30		30	30	30	30	30	30	30
TG	**r**	0.180	0.042	0.062		0.077		0.044	0.162	-0.081	0.024	0.087	-0.119	-0.029
	*p*	0.350	0.827	0.751		0.690		0.819	0.400	0.674	0.902	0.653	0.537	0.881
	**n**	29	29	29		29		29	29	29	29	29	29	29
Urea	**r**	-0.003	0.046	-0.090		-0.097		-0.120	-0.215	0.119	-0.178	-0.168	0.204	-0.135
	*p*	0.987	0.811	0.642		0.615		0.536	0.262	0.539	0.356	0.384	0.287	0.486
	**n**	29	29	29		29		29	29	29	29	29	29	29
Creatinine	**r**	0.000	0.040	0.072		-0.157		-0.060	-0.075	0.098	-0.018	-0.162	0.094	-0.127
	*p*	0.999	0.838	0.710		0.415		0.756	0.698	0.613	0.927	0.401	0.627	0.512
	**n**	29	29	29		29		29	29	29	29	29	29	29
Urine albumin/ creatinine ratio	**r** *p*	0.000 0.998	-0.044 0.819	-0.112 0.555		0.082 0.665		0.150 0.430	-0.195 0.302	-0.053 0.780	-0.167 0.378	-0.030 0.873	-0.054 0.775	-0.075 0.694
	**n**	30	30	30		30		30	30	30	30	30	30	30

SSI= signal strength index; FT= foveal thickness; PT= parafoveal thickness; CC = coriocapilar; FAZ= foveal avascular zone; TSVD= total superficial vascular density; FSVC= foveal superficial vascular density; PSVD= parafoveal superficial vascular density; TDVD= total deep vascular density; FDVD= foveal deep vascular density; PDVD= parafoveal deep vascular density; r= Pearson’s correlation coefficient; *p*= p value; BCVA= best-corrected visual acuity; FBG= fasting blood glucose; HbA1C= glycosylated hemoglobin; TC= total cholesterol; TG= triglyceride.

## DISCUSSION

Dilated funduscopy is the standard examination used to screen for DR. OCTA is a rapid, noninvasive method that provides details of the retinal microvasculature. We hypothesized that the latter technique might detect early changes that are not observable on clinical or angiographic examinations because microvascular changes occur before the development of clinically detectable retinopathy^(12)^. On first examination, no significant differences were found between the two study groups, with the exception of the CC flow.

Previous studies have reported choroidal changes. The first qualitative evaluations were based on histology, indocyanine green angiography, and Doppler flowmetry techniques; the abnormalities described in those studies included endothelial loss, microaneurysms, di lations and CC obstructions, remodeling with increased vascular tortuosity, and areas of nonperfusion and neovascularization^(13)^. Other structural abnormalities in diabetic choroidopathy have been explored with the advent of OCT technologies^(13)^. The most recent reports have explored choroidal thickness. Several authors have reported that choroidal thickness is decreased in diabetics; this decrease is associated with DR retinal sequelae because the choroid is responsible for oxygenation and ensures the delivery of adequate nutrition to the external retina and retinal pigment epithelium. Damage to the choroid may increase retinal susceptibility to hypoxia and ischemia^(13-17)^. Some of those studies reported that choroidal thickness decreases even in the absence of DR, suggesting that the thinning and reduced choroidal flow could be a primary occurrence^(14,16)^. However, other studies have reported alternative findings because progressive choroidal thickening occurred in line with DR progression^(13,18,19)^, and this development also was reported in patients with diabetes without retinopathy^(20)^.

Impaired choroidal flow with and without DR during OCTA^(8)^ and reduced CC density were also observed among diabetic patients without retinopathy^(21)^. However, it remains unclear whether these choroidal changes are primary responses to or independent of DR^(13)^.

In the current study, CC flow areas of 22.3 ± 4.6 and 22.6 ± 3.9 mm^2^ were observed in the control group and among patients with diabetes, respectively (p=0.017). The variation in CC flow between the groups was small, and the clinical relevance of this variation remains questionable. However, to our knowledge, the current study is the first to quantitatively evaluate the CC flow area in patients with diabetes without DR; thus, the lack of data in the literature regarding this parameter prevents a comparative evaluation. The current findings may be related to reports of choroidal thickening. However, the current study did not identify a correlation between the BCVA and changes in the CC flow (p=0.783).

Recent reports have reported that increases in the FAZ are an indicator of DR progression and have described the changes associated with several stages of retinopathy, as determined by OCTA^(9,12)^. Using ImageJ software and FAZ manual demarcation, SCP and DCP widening was reported in diabetic eyes without DR, and OCTA was suggested to be able to detect eyes at a higher risk of developing DR^(9)^. Further, with the manual demarcation of the FAZ and using MATLAB (MathWorks, Natick, MA, USA) for analysis, another group obtained a similar result in a sample of patients with diabetes without retinopathy^(22)^. No significant differences in the FAZ of the SCP were observed between the two study groups of the current sample. This difference in findings between reports may be a result of the different methods used to measure the FAZ; in the current study, we used the automatic measurement function provided by the device using a nonflow area tool. Conversely, the previously cited studies performed manual measurements of the images obtained by the OCTA Avanti RTVue with the aid of other software. The current results agree with those of a 2017 report that compared the FAZ of the SCP of 71 diabetics without DR with that of 67 healthy individuals using methodology similar to ours^(21)^.

The VD was evaluated in DR using different methods and devices; several publications have reported reduced VDs in both plexuses with the progression of DR^(7,10,23)^. It is assumed that VD can predict the severity of DR with relative specificity and sensitivity^(24)^.

Few researchers have evaluated VD in the absence of retinopathy. In the current study, no significant differences were observed when comparing the VDs between healthy and diabetic eyes, even when considering the SCP and DCP as well as the foveal and parafoveal areas. However, some studies have reported contradictory findings, i.e., reduced VDs at the DCP in patients with type 1 diabetes, suggesting that a decrease in VD might be an early process in type 1 DM that occurs initially in the DCP^(25)^. A 2019 study reported increased perfused capillary density (PCD) values in patients with types 1 and 2 diabetes without DR and a reduction in the PCD in the presence of DR. Theoretically, increased PCD occurs due to the autoregulatory response to increased metabolic demand in the stage before clinical DR and is attributed primarily to vascular dilation. Those researchers defined PCD as a perfused capillary area divided by the corresponding analyzed area after the subtraction of noncapillary blood vessel areas. In contrast to our analysis, which included capillaries and noncapillary vessels, that study found no significant difference in the noncapillary blood vessel density between the healthy controls and diabetics. The authors suggested that the inclusion of noncapillary vessels, as in some previous studies, may reduce the sensitivity of the analysis and overlook the upward inflection in the PCD before its decline as DR progresses; thus, the PCD may have use as a biomarker. However, the same authors reported that their study was limited because they did not include clinical and laboratory data in the analysis^(26)^.

DRCR.net researchers compared the central macular thickness values in 97 patients with diabetes who had either no or minimal DR with individuals without diabetes. The results suggested that DM is not associated with significant changes in the macular thickness in the absence of DR because there was no significant difference between the groups^(4)^. Likewise, the current study did not reveal any differences between the two groups in either the foveal or parafoveal thickness values.

Several studies have reported that better glycemic levels were associated with fewer retinal complications^(4)^. HbA1c levels that are indicative of DR vary between 6.5% and 6.1%^(27)^. In our sample, the mean HbA1c level was 7.8% ± 2.5%, which is higher than normal values; this may justify some of the current findings. We observed a significant negative correlation between HbA1c and FBG levels and the foveal VD in SCP and DCP; this is in contrast to the results published in 2018 that did not associate the VD in the plexus with the HbA1c levels in diabetics without DR^(21)^. Others have reported a similar negative correlation between HbA1c levels and VD in the DCP^(28)^; however, this was observed in a population of patients with DR. The current findings suggested that VD changes in response to glycemic changes can occur earlier than previously anticipated.

The current study identified a positive correlation between the CC flow area and FBG levels (p=0.034). We did not identify any published studies that examined the relationship between these clinical variables. However, a correlation was observed between the HbA1c levels and increased choroid thickness in a group of patients with diabetes who were hospitalized and underwent acute glycemic control for two weeks; the same results were not found in association with the FBG levels^(29)^. Another report also described the lack of a correlation between choroid thickness and FBG levels in diabetic patients without DR, but those subjects had a mean glucose level of 124.0 ± 29.0 mg/dL, which is lower than the values obtained in our population (167.1 ± 65.0 mg/dL)^(15)^.

We did not observe a correlation between the AngioAnalytics findings for the lipid profiles and renal function. The literature has a dearth of information regarding the association between these two risk factors and OCTA findings. A 2017 report associated hypercholesterolemia and renal impairment in diabetics with and without retinopathy with reduced VD^(23)^. Other researchers described an association between increased serum lipid levels and increased choroidal thickness in 322 eyes of patients without diabetes; the authors reported that this association was a result of choroidal atherosclerotic changes^(30)^. A third study found no significant correlations between the superficial and deep VDs and serum creatinine levels in diabetics without DR^(21)^.

Various researchers have agreed that quantitative measurements of retinal and choroidal microvascular changes are important tools for assessing DR progression and responsiveness to therapy and that these methods may elucidate the association between DR and visual acuity changes in diabetics^(8,24)^. Some authors have also suggested that OCTA could be used to diagnose preclinical DR and, along with primary care physicians, could better ensure an ideal clinical/ophthalmologic follow-up of these patients that includes glycemic control and monitoring of serum lipids and blood pressure^(21)^. However, based on our data, we concluded that OCTA may not be an ideal tool to ensure an early diagnosis of DR and that the technology cannot be used to replace clinical funduscopic examinations. No significant differences were found in the retinal parameters evaluated in the current study. Thus, we cannot definitively conclude whether there are alterations in CC flow before the onset of DR since the differences between the two study groups were minimal and apparently not clinically relevant. We also recognize that 3x3-mm angiograms provide better resolution and that swept-source OCT is preferable for analyzing the CC. However, the current study provided new information on possible changes in the CC of patients with diabetes, and the results suggested that OCTA may be a better tool to calculate the CC flow area, which, to the best of our knowledge, has not yet been described in the literature. Future studies should assess the correlation between the CC flow area and changes in choroidal thickness. Conversely, the findings corroborate the idea that glycemic control should be the first clinical parameter assessed in DR screening.

The current study has some limitations: the sample size was small and a cross-sectional design was used. Larger studies with longer follow-up periods may identify more changes in the OCTA findings described in the study population and confirm the current findings.
